# Development of an attention-touch control for manual cervical distraction: a pilot randomized clinical trial for patients with neck pain

**DOI:** 10.1186/s13063-015-0770-6

**Published:** 2015-06-05

**Authors:** M. Ram Gudavalli, Stacie A. Salsbury, Robert D. Vining, Cynthia R. Long, Lance Corber, Avinash G. Patwardhan, Christine M. Goertz

**Affiliations:** Palmer Center for Chiropractic Research, Palmer College of Chiropractic, 741 Brady Street, 52803 Davenport, IA USA; Musculoskeletal Biomechanics Laboratory, Edward Hines Jr. VA Hospital, Hines, IL and Department of Orthopedic Surgery & Rehabilitation, Stritch School of Medicine, Loyola University Chicago, Chicago, IL USA

**Keywords:** Adverse effects, Chiropractic, Clinical trial, Control groups, Forces, Traction, Manual therapy, Musculoskeletal manipulations, Neck pain, Outcome assessments, Traction

## Abstract

**Background:**

Manual cervical distraction (MCD) is a traction-based therapy performed with a manual contact over the cervical region producing repeating cycles while patients lie prone. This study evaluated a traction force-based minimal intervention for use as an attention-touch control in clinical trials of MCD for patients with chronic neck pain.

**Methods:**

We conducted a mixed-methods, pilot randomized clinical trial in adults with chronic neck pain. Participants were allocated to three traction force ranges of MCD: low force/minimal intervention (0-20 N), medium force (21-50 N), or high force (51-100 N). Clinicians delivered five treatments over two weeks consisting of three sets of five cycles of MCD at the C5 vertebra and occiput. Traction forces were measured at each treatment. Patient-reported outcomes included a pain visual analogue scale (VAS), Neck Disability Index (NDI), Credibility and Expectancy Questionnaire (CEQ), and adverse effects. A qualitative interview evaluated treatment group allocation perceptions.

**Results:**

We randomized 48 participants, allocating an average of five each month. Forty-five participants completed the trial with three participants lost to follow-up. Most participants were women (65 %) and white (92 %) with a mean (SD) age of 46.8 (12.5) years. Mean traction force values were within the prescribed force ranges for each group at the C5 and occiput levels. Neck pain VAS demonstrated a benefit for high traction force MCD compared to the low force group [adjusted mean difference 15.6; 95 % confidence interval (CI) 1.6 to 29.7]. Participants in the medium traction force group demonstrated improvements in NDI compared to the low force group (adjusted mean difference 3.0; 95 % CI 0.1 to 5.9), as did participants in the high traction force group (adjusted mean difference 2.7; 95 % CI -0.1 to 5.6). CEQ favored the high force group. Most low force participants correctly identified their treatment allocation in the qualitative interview. No serious adverse events were documented.

**Conclusions:**

This pilot study demonstrated the feasibility of a clinical trial protocol and the utility of a traction-based, minimal intervention as an attention-touch control for future efficacy trials of MCD for patients with neck pain.

**Trial registration:**

ClinicalTrials.gov NCT01765751 (Registration Date 30 May 2012)

## Background

Chronic neck pain is a common musculoskeletal complaint associated with radiating symptoms to the head, upper extremities or thoracic regions, muscle stiffness, sensorimotor dysfunction, headache, vertigo, and psychological complaints [1–4]. The 12-month prevalence of neck pain in adults is estimated at 30 % to 50 % [5], with symptoms reported more frequently by women and middle-aged people [6–8]. Health service utilization among patients with neck pain is substantial, with 10.2 million visits to US healthcare facilities registered in 2007 [9].

Pharmacological and surgical interventions for chronic neck pain are available [9, 10], but many procedures are costly, leading to rising healthcare expenditures for this condition [11, 12]. In addition, non-steroidal anti-inflammatory medicines, opioids, spinal injections, and spine surgery have high-risk side effects [13–15] and may deliver only marginal improvements to patients’ clinical outcomes, work-related productivity and quality of life measures [11, 12]. Preliminary evidence suggests that manual therapies may provide similar benefits to patients with chronic neck pain as conventional medical treatments, with better tolerance and safety profiles [16–19]. However, a recent systematic review recommends additional studies to determine the efficacy of specific manual therapies to clinical outcomes in this patient population [20].

Clinical studies involving mechanical or manual therapies have demonstrated mixed clinical outcomes in patients with chronic neck pain [21–25]. These conflicting results are likely due to varying study methodologies and quality levels, preventing a conclusive determination of the efficacy of mechanical traction and other manual therapies for the relief of neck pain [26–31]. To improve the quality of such studies, recent systematic reviews have encouraged researchers to develop sham or minimal interventions to serve as control groups for clinical trials of manual treatments for neck pain [17, 32]. Toward this aim, our team conducted a series of basic science and clinical studies to develop a minimal intervention to serve as an attention-touch control group for efficacy trials of manual cervical distraction (MCD).

MCD is an intermittent traction procedure that differs from mechanically based traction therapies. MCD is delivered using a manual contact over different vertebral levels of the cervical spine in slow, repeating phases while the patient lies prone, whereas mechanical traction uses a head harness attached to weights or computer controlled tensioning equipment with patients in supine or upright positions [33]. MCD is hypothesized to create intersegmental separation at targeted vertebral levels, which contributes to generating a therapeutic effect [34]. Although MCD is used in the care of persons with neck pain [35–39], no published clinical trials have evaluated the efficacy of this therapy, in part due to the lack of adequate sham treatments or control intervention.

Our investigative team conducted a series of preliminary studies to develop a sham treatment or minimal intervention to serve as an attention-touch control for randomized controlled trials of MCD. Our objective in these studies was to identify cervical traction force ranges that demonstrated no intradiscal pressure decreases, but that patients with neck pain believed to be a credible treatment. In the first preliminary study, Gudavalli and colleagues (2013) confirmed that MCD decreased cervical spine intradiscal pressures in cadaveric specimens, even with traction forces as light as 20 N [40]. We then evaluated the perceived sensations of participants undergoing MCD to identify force ranges that participants classified as non-therapeutic, possibly therapeutic, and definitely therapeutic, with patients confirming 25.5 N as a possibly therapeutic level of traction force [41]. Based on the results of these preliminary studies, we concluded that developing a low-force minimal intervention will be a better choice than a sham treatment. We next developed a force feedback technology [42] to allow clinicians to deliver MCD within distinct traction force ranges, and tested a training and certification process using this technology [43]. These data led us to conclude that MCD could be delivered consistently within three different traction force ranges [44]. Our team then assessed clinician proficiency in delivering specified traction forces during a pilot randomized clinical trial (RCT) of MCD [45, 46].

The primary objective of this investigation was to evaluate a traction force-based, minimal intervention for patients with neck pain to use as an attention-touch control in future fully powered randomized trials studying MCD. This article also reports additional findings from this pilot RCT, including the feasibility of our participant recruitment plan and treatment protocol, the results of patient-reported outcomes (PROs), traction forces delivered, and patient perceptions of the three force-based treatment groups. Our presentation follows recent commentaries on reporting results of clinical trial pilot studies [44, 47].

## Methods/design

This pilot RCT with a nested qualitative study compared three traction force ranges of MCD in 48 adults with chronic neck pain. Participants were randomized to one of three study groups: 1) low force/minimal intervention [0-20 Newtons (N)], 2) medium force (21 N-50 N), or 3) high force (51 N-100 N) MCD. At each treatment visit, participants received three sets of five repetitions of traction with a neutral head position, delivered within the allocated force range and at two contact points (C5 and occiput). Participants completed five study visits over two weeks, with PROs collected at baseline visit 1 (BL1) and before receiving treatment on the first and fifth study visits. Primary PROs included the Visual Analogue Scale (VAS) for neck pain and the Neck Disability Index (NDI). Secondary PROs included the Patient Reported Outcomes Measurement Information System (PROMIS-43) general health status scale and patient satisfaction. Patient perceptions of study treatments and adverse events also were assessed. We evaluated participant blinding to their study intervention in a qualitative interview conducted after receiving their fifth MCD treatment on the last study visit.

### Ethical considerations

The Palmer College of Chiropractic Institutional Review Board approved the study protocol and human subjects protection procedures for this trial (Approval #2012G151, date 1 November 2013). Study coordinators provided an overview of the research procedures and played a video-recording that demonstrated the study procedures. The RCT was not identified as a pilot study in the consent; however, the written document identified all required elements for informed consent [48]. All participants read and signed the written consent document before the BL1 evaluation.

### Participant eligibility and recruitment

We recruited participants of both genders, 18- to 70-years old using strategies that were successful in previous studies, including direct mail postcards, media releases, and internet postings [49]. Our targeted sample size was 45 participants. Participants completed a multi-stage eligibility determination process that included a telephone screen; in person informed consent process, questionnaires, clinical interview and examination by a Doctor of Chiropractic (DC); and a panel-based case review [50]. Eligible participants had self-reported neck pain or neck-related upper extremity pain of at least four weeks duration ranging in intensity from 3 to 7 on an 11-point (0 to 10) numerical rating scale (NRS) on the telephone screen [51]. Participants were not excluded for previous treatment of this episode of neck pain. However, because our primary aim was to develop a minimal intervention for MCD, participants could not have had any previous experience receiving flexion-distraction technique to the cervical spine. Table 1 displays each eligibility criterion with the corresponding rationale. Participant progress through the trial was recorded via a secure, password-protected, web-based tracking system according to CONSORT 2010 standards [52].

**Table 1:** Eligibility criteria

Inclusion criteria	Rationale
Neck or neck-related upper limb pain consistent with Quebec Task Force (QTF) classifications 2-4 [75]	Study intervention was designed to treat radiating neck pain (proximal or distal extremity) with or without neurological signs
Naïve to manual cervical distraction procedures to cervical spine region	Treatment credibility assessment requires unfamiliarity with study interventions
Exclusion criteria	Rationale
Neck pain without radiation (QTF classification 1) [75]	Intervention designed to treat radiating neck pain
Presumptive compression of a nerve root on roentgenogram (i.e, instability fracture) (QTF classification 5)	May require individualized treatment not available in trial
Compression of spinal nerve root confirmed with imaging (QTF classification 6)	May require individualized treatment not available in trial; condition may limit interpretation of study measurements
Spinal stenosis (QTF classification 7)	May require individualized treatment not available in trial; condition may limit interpretation of study measurements
Postsurgical status <6 months (QTF classification 8)	May require individualized treatment not available in trial; condition may limit interpretation of study measurements
Postsurgical status >6 months (QTF classification 9)	May require individualized treatment not available in trial; condition may limit interpretation of study measurements
Chronic pain syndrome (QTF classification 10)	Care needed is outside study scope
Other diagnoses from visceral disease, metastasis, etc*.* (QTF classification 11)	Care needed is outside study scope
Inflammatory arthritis in the cervical spine: i.e*.*, rheumatoid arthritis, systemic lupus erythematosus	Study treatments are not intended for these conditions
Neurological (spinal) instability in cervico-thoracic spine	Treatment needed is outside study scope
Tumors, within or adjacent to the cervico-thoracic spinal canal	Treatment needed is outside study scope
Arnold Chiari malformation	May require referral, additional evaluation or treatment outside study scope
Spinal joint hypermobility, such as: Marfan syndrome, Ehlers-Danlos syndrome, osteogenesis imperfecta	May interfere with data collection and interpretation
Advancing neurologic deficits	Treatment needed is outside study scope
Sequestered intervertebral disc or loose body within the cervical spinal canal, lateral recess, or intervertebral foramen	Safety precaution
Fusion (single or multisegmental) of the cervical vertebrae	Joint distraction and intervertebral disc pressure change are hypothesized as principal therapeutic mechanisms
Safety precaution (e.g*.*, unable to ambulate within clinic, dizziness, weight beyond treatment table limits [300 lbs])	Safety precaution
Unable to tolerate study procedures	Safety precaution
Simultaneous management for a condition compromising ability to deliver study treatment or assess health status	Safety precaution and may present an undue scheduling burden
Suspicion of alcohol or drug abuse	May interfere with data collection, ability to comply with study protocol, and requires referral
Cognitive or memory impairment	May prohibit informed consent or compromise safety due to potentially reduced comprehension or compliance with study procedures
Referral for evaluation/management of other condition(s) or neck pain diagnosis	Safety precaution and advanced diagnostic testing outside study scope
Compliance concerns	Transportation issues, scheduling conflicts, etc*.* may compromise adherence to study protocol

### Setting

This study was conducted at the Palmer College of Chiropractic, Palmer Center for Chiropractic Research, in Davenport, IA, USA. The dedicated research clinic is a 250 square meter (2,700 square foot) facility with consultation, exam, and treatment rooms, clinical biomechanics laboratory and radiology suite. The clinic is staffed with research clinicians who are licensed DCs, study coordinators, and a research participant coordinator. The laboratory is staffed by biomechanics faculty and research assistants. Participants completed data collection and interviews in a private consultation room. Treatments and biomechanical measurements took place in a single room equipped with the treatment table and computerized biomechanical instrumentation, and staffed with up to three research personnel.

### Study visits

Volunteers were screened by telephone and, if eligible, scheduled for a baseline visit (BL1) that included the informed consent process, baseline measures, an interview by a study coordinator, medication and health history review, an eligibility exam by a DC and, if indicated, a cervical radiographic evaluation. BL1 eligible participants were presented by the examining clinician to the case review panel, which rendered an eligibility determination [50]. The case review panel consisted of a co-investigator, project manager, clinicians, and study coordinators. Eligible participants at a second baseline visit (BL2) were assigned at random to one of three intervention groups and completed study visit 1 (SV1). Participants completed five study visits scheduled at least one day apart over two weeks with no more than three study visits during any single week. Study visits included questionnaires, clinical assessments, group-specific interventions, and specified research measurements.

### Treatment group allocation

Participants were allocated to one of three treatment groups with a predetermined blocked randomization scheme with random block sizes of three or six created with computerized random numbers in a 1:1:1 ratio. A research clinician entered the participant identification number into the study-specific, web application that generated the coded treatment group, which was known only to the clinician. This web application algorithm concealed future group assignments.

### Intervention

**Fig. 1:**
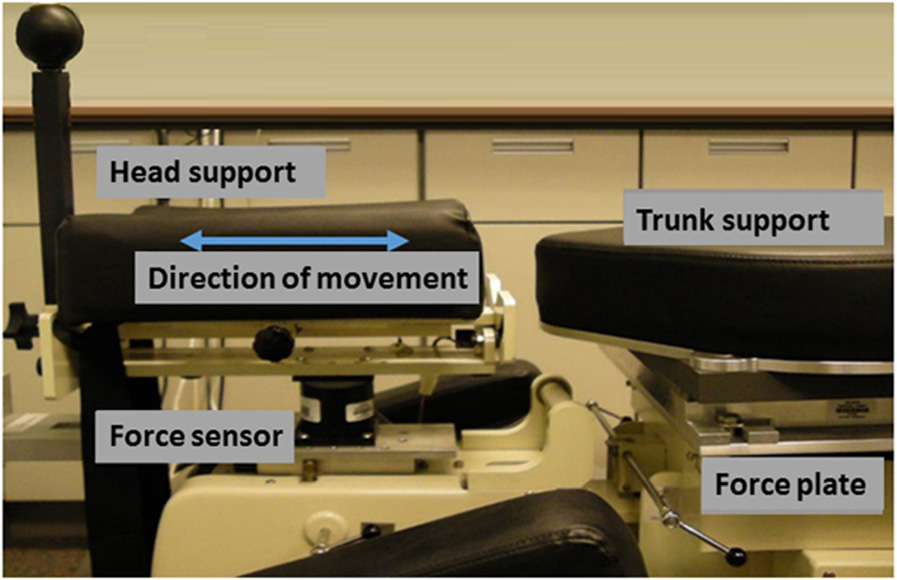
Head and thorax support sections of instrumented manual distraction treatment table used in the study

MCD is a form of low velocity variable amplitude spinal manipulation [34]. Figure 1 depicts the instrumented Cox Flexion-Distraction Table (Version 7, Haven Innovations Inc., Detroit, MI, USA) used for all trial treatments. The table includes a moveable headpiece that allows for guided head movement in multiple directions; however, only cephalic movement (traction) with the head in a neutral position was used in this study. During MCD, the participant laid prone on the treatment table while the clinician gently grasped the posterior aspect of the participant’s neck with a broad contact (contact hand) between the thumb and index finger at a specific vertebral level (standardized at C5 and occiput). With the opposite hand, the clinician held a control handle attached to the headpiece. Using the contact hand, the clinician exhibited superior traction while ensuring gentle headpiece movement via the control handle. The weight of the participant’s head and the light pressure from the manual contact was sufficient to maintain contact with the headpiece during the traction movement. The procedure produced a rhythmic, localized distractive movement lasting one to three seconds per repetition. Based on the findings of a preliminary study of patient perceptions of MCD traction forces [41], we limited cervical traction forces to less than or equal to 20 N in the low force group, between 21 N and 50 N in the medium force group, and between 51 N and 100 N in the high force group. Dosing was limited to three sets of five repetitions with contact over the C5 cervical vertebra and three sets of five repetitions with contact on the occiput. To insure inter- and intra-clinician proficiency in treatment implementation [46], we conducted pre-trial training sessions and monthly clinician recertification on traction force delivery using a process described elsewhere [43].

**Fig. 2:**
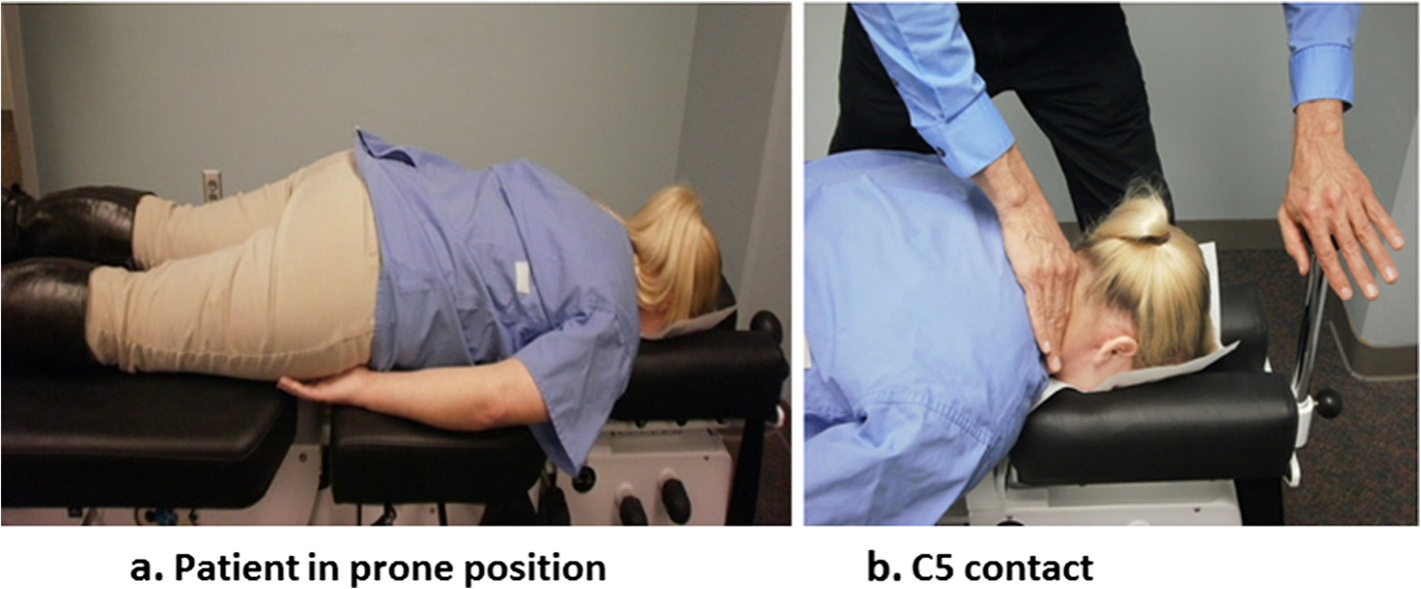
Photographs showing (**a**) participant lying prone on treatment table and (**b**) clinician hand contact at C5 vertebral level

Participants wore a collarless shirt and placed their hands under their thighs instead of on the table hand rests, if able, to assure consistent positioning and force measures between participants and study visits (Fig. 2). All moveable sections of the table were locked in place except for the axial (cephalic) movement mechanism of the headpiece. We used only traction with a neutral head position (Cox protocol 1) [34] to standardize treatment delivery between participants and study visits.

### Measurements

Additional measures were as follows. A verbal 0 to 10 numerical rating scale for pain was used at the telephone screen for eligibility determination due to its ease of administration for data collection [51]. We collected demographics at the phone screen and at the BL1. We assessed participants at BL1 for severe depression using the Beck Depression Inventory [53]. Study coordinators completed a medication checklist with participants at SV1 and SV5. PROs and questionnaires to assess treatment credibility and participant expectations were collected on participant-administered paper forms at BL1 and before treatment delivery at SV1 and SV5. Cervical traction forces were collected during each treatment. Cervical range of motion was measured at SV1 and SV5. A qualitative interview was completed after other study procedures on SV5. There were no follow-up assessments.

### Patient-reported outcomes

PROs included the Visual Analogue Scale (VAS), Neck Disability Index (NDI), the Patient Reported Outcomes Measurement Information System (PROMIS-43) general health status scale, and patient satisfaction. The VAS is a single-item, unidimensional measure for pain with excellent metric properties, easy administration and scoring that is commonly used in pain-related research [51]. Our anchors for current neck pain were ‘no pain’ to ‘worst imaginable pain’ using a 0 to 100 mm scale, which allowed for more sensitive assessment of pain intensity compared to the NRS due to its increased number of response levels [54]. Score interpretations for the VAS include the following cut points: no pain (0 to 4 mm), mild pain (5 to 44 mm), moderate pain (45 to 74 mm), and severe pain (75 to 100 mm) [55]. The NDI is a 10-item questionnaire modified from the Oswestry Disability Index for low back pain [56, 57]. The NDI is a reliable and valid measure of disability due to neck pain, and responsive for measuring change on a scale of 0 to 50. Following the Initiative on Methods, Measurement and Pain Assessment in Clinical Trials (IMMPACT) recommendations for chronic pain clinical trials, we measured several additional core outcomes, including physical and emotional function [58] using the PROMIS-43 to evaluate pain interference, physical function, fatigue, and sleep disturbance [59]. Participants rated their satisfaction with various elements of the study (e.g., treatments received, clinicians) using a 5 category response from 1 (strongly satisfied) to 5 (strongly dissatisfied) with categories collapsed into satisfied (categories 1 and 2) or not satisfied (categories 3, 4 or 5) for interpretation.

### Cervical traction forces

A research assistant trained in biomechanical measurements recorded cervical traction forces during all study visits. The treatment table was modified to include force plates and motion sensors (Model 4060-1000, Bertec Inc., Columbus, OH, USA) to measure traction forces and table movement. Force plates were connected to a computer through an analog-to-digital converter. Motion Monitor Software (Version 7.1, Innovative Sports Training Inc., Chicago, IL, USA) collected data from the force plates and motion sensors at a sampling rate of 100 Hz. Data were exported to text file using the export function of the software. Custom written Matlab software (Version 7, Mathworks Inc., Natick, MA, USA) read the exported data and graphed the forces as a function of the duration of treatment. A research assistant then used starting and end time points on the force-time graph to extract the peak traction forces between these time points. Peak forces data were exported from Matlab to an MS-Excel 2010 file (Microsoft Corporation, Redmond, WA, USA) and the average peak force in N was computed.

### Credibility and Expectancy Questionnaire

Participants’ perceptions of the study treatments were compared across groups using the Credibility and Expectancy Questionnaire (CEQ) [60, 61]. The CEQ credibility factor relates to how logical or believable a treatment is for the patient, whereas the expectancy factor refers to the patient’s beliefs about improvements that might be achieved from the treatment [61]. CEQ scores for both the credibility and expectancy factors range from 3 to 27, with higher scores indicating greater belief in treatment credibility or expectancy for clinical improvement. Psychometric evaluations of the CEQ in varying populations demonstrated high internal consistency (standardized α = 0.79 to 0.90 expectancy; Cronbach’s α = 0.81 to 0.86 credibility; standardized α = 0.84 to 0.85 whole scale), good inter-item correlations (expectancy ranging from 0.53 to 0.85; credibility ranging from 0.62 to 0.78) and high test-retest reliability over one-week (0.82 expectancy; 0.75 credibility) [61]. Participants completed the CEQ at the BL1 after reading the consent document and viewing the introductory video about MCD, at the SV1 after receiving the MCD treatment for the first time, and at the SV5 before receiving their final treatment.

### Qualitative interviews

As this was a pilot study evaluating a novel minimal intervention for use as an attention-touch control in future clinical trials, we assessed participants’ perceptions of study treatments through standardized qualitative interviews, as recommended in a previous placebo-controlled trial of manual therapies [62]. One investigator (SAS) who has expertise in qualitative methods conducted all interviews with participants after their completion of the PROs, biomechanical measures, and MCD treatment at SV5. A professional transcriptionist transcribed the interviews verbatim. The interviewer was blinded to treatment group until after a question on treatment group assignment was asked during the interview schedule. Qualitative data were imported into NVIVO software (Version 9, QSR International Pty Ltd., Doncaster, Victoria, Australia) to facilitate data management and qualitative content analysis [63]. We appraised whether participants correctly identified their treatment group assignment (low force, medium force or high force) and their reasons for attributing treatment allocation to this group. Responses were broken down into discrete statements, marked with codes, and grouped into themes based on recurring patterns [64].

### Data management

We have described our data management procedures elsewhere [65, 66]. All data collection forms had unique participant identification numbers. Data forms were stored in locked filing cabinets or on a secure, password-protected server accessible only by study personnel. Data were double-key entered into computer databases that contain range and logic checks for accuracy. Electronic data were stored on an internal Microsoft SQL Server secured with Secure Socket Layers (SSL). Biomechanical data were stored on a secured network drive. Analytic and qualitative files were maintained on a secure, password-protected server.

### Sample size

This pilot RCT was not a powered study. We targeted a sample size of 15 per group (n = 45) to provide adequate participant contact to evaluate study protocol feasibility and recruitment strategies and to assess the believability characteristics of the three traction force-based treatments. We also estimated group differences in mean changes and standard deviations in the outcome measures. These aspects of the study protocol were selected as key to informing the design of a full‐scale efficacy trial of MCD.

### Statistical methods

Participants who had non-missing data for the primary outcomes were analyzed according to their original treatment assignment. We performed quantitative data analyses using SAS/STAT version 9.3 (SAS Institute, Inc., Cary, NC, USA). Change in PROs from BL1 to SV5 (before biomechanical measurements and treatment) were compared between groups with analysis of covariance (ANCOVA), adjusting for the BL1 value of the respective variable centered at its mean. We then entered baseline variables that were imbalanced among groups, as well as a variable differentiating if the intervention was aided by visual feedback, into the models one at a time. Only variables significant at the 0.10 level were kept in the final models. Adjusted within group changes and between group differences in mean change and 95 % confidence intervals based on the final ANCOVA models are presented.

### Blinding protocol

Clinicians were not masked to group assignment, but were blinded to PROs. Study coordinators, examining clinicians, investigators and analysts were blinded to group assignment. Biomechanical technicians were blinded to group assignment but not traction forces, while research assistants completing data reduction were blinded to group assignment. The qualitative interviewer was blinded to group assignment during the exit interview until the participants were asked a specific question on treatment group assignment, at which point the participant and researcher were unmasked to treatment group. However, the qualitative researcher was masked to treatment group during data coding. Participants were masked to group assignment during the trial until questions on the exit interview specifically asked them to guess their treatment group assignment.

### Safety, protocol deviation and compliance monitoring

Recent articles have called for more consistent, in-depth and standardized monitoring of adverse events related to manual therapies [67–71]. Toward that aim, we carefully monitored adverse events (AE) and serious adverse events (SAE) using an active surveillance process described elsewhere [65, 66]. AEs were defined as any untoward medical occurrence that may present itself during the conduct of the study which may or may not have a causal relationship with the study procedures [72]. SAEs were defined as any adverse experience occurring during treatment that results in any of the following outcomes: death, life-threatening adverse experience, hospitalization, persistent or significant disability or incapacity, or a congenital anomaly or birth defect [72]. Clinicians collected AE data by directly querying participants about any new injuries, side effects or symptoms at each visit. Positive responses initiated the completion of an Adverse Event Report, with all reported AEs and SAEs graded on three criteria: 1) severity; 2) expectedness; and 3) relatedness to the study interventions. The informed consent document disclosed muscle soreness or stiffness, light-headedness or dizziness, headache and exacerbation of neck pain symptoms as AEs experienced by patients receiving manual therapy for neck pain [73, 74].

Protocol deviations were tracked using a study-specific, web application that included the date of occurrence, participant(s) involved, detailed event notes, and investigator response. Participant compliance was tracked through the scheduling database that included details on missed appointments and study dropouts.

## Results

### Participant flow and recruitment feasibility

**Fig. 3:**
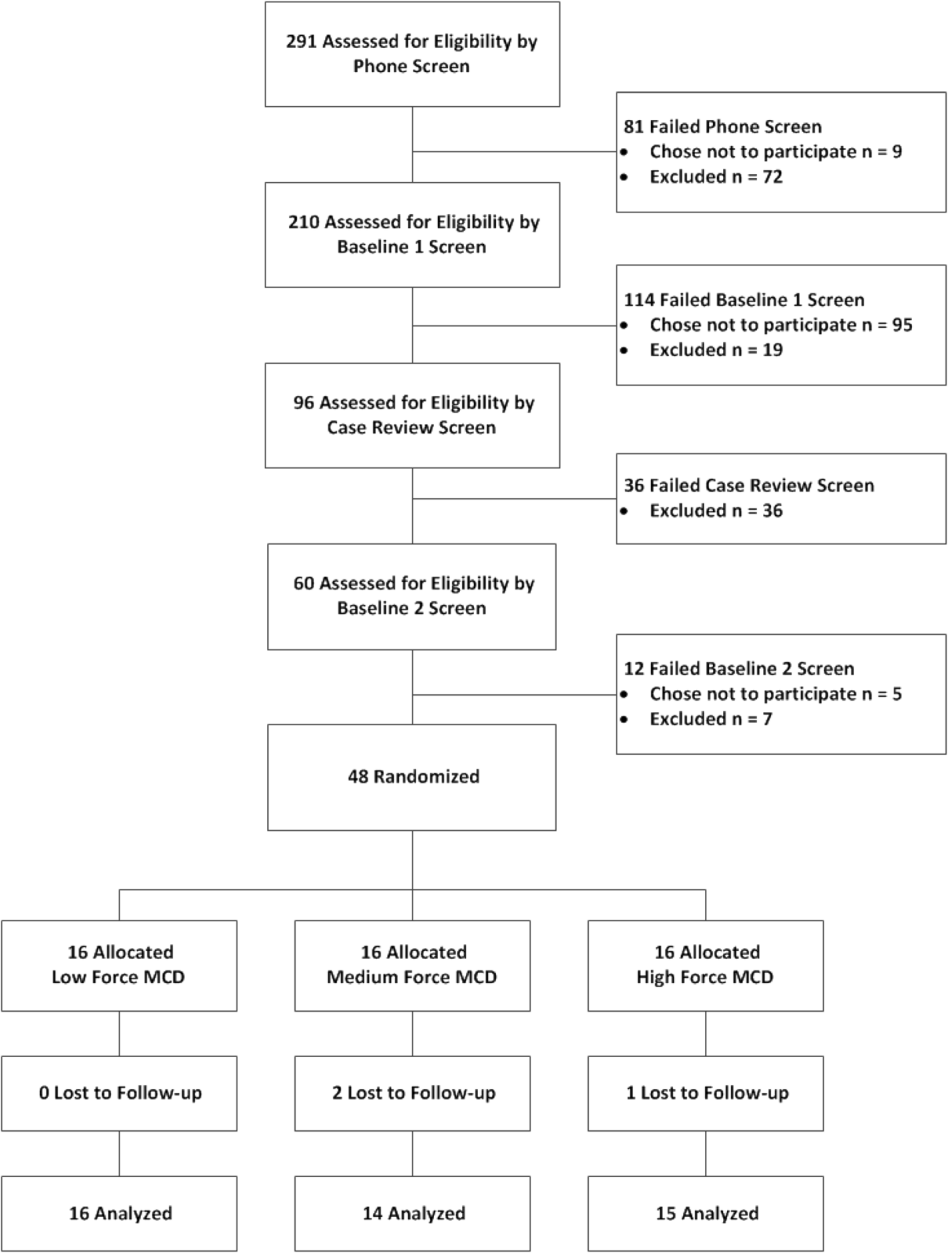
Participant flow diagram

Figure 3 presents the participant flowchart. We completed 291 telephone screens, with 210 participants eligible for the BL1 clinic screen. Ninety-six participants were presented at case review, with 60 participants eligible for the BL2 clinic screen. In total, 109 participants chose not to participate in the trial and 134 participants were excluded. The most common reasons participants did not enroll in the study were non-attendance at a scheduled appointment (n = 43), time or other outside commitments (n = 30), not interested (n = 16), not willing to stop treatment from another provider (n = 5), other health concern (n = 4), neck condition not requiring treatment at this time (n = 3), not willing to receive study treatments (n = 2), or non-specified reasons (n = 6). The most common exclusions were for a neck pain rating of ≤2 on the NRS (n = 37), a neck pain rating of ≥8 on the NRS (n = 32), current litigation (n = 10), inability to tolerate study procedures during BL1 screening (n = 9), the need for referral or concurrent treatment identified during BL1 screening (n = 6), Quebec Task Force classification of neck symptoms outside the ratings of 2, 3, or 4 (n = 6), multiple exclusions (n = 17), or other exclusions (17). We randomized 48 participants to treatment, allocating an average of five participants each month (range 1 to 10 participants/month). Forty-five participants completed the trial and three participants were lost to follow-up (two medium force participants and one high force participant). We were unable to ascertain the reasons these three participants dropped out of the study. Only four participants missed any of the five scheduled treatment visits; of these, three participants were those who later withdrew from the study, missing two, three, and four treatments, respectively. We opted not to perform study treatments or biomechanical tests on one participant who experienced an adverse event (described below) following the first study visit. This participant did not attend two study visits, but completed all outcome measures.

### Baseline demographic and clinical characteristics

Table 2 depicts the BL1 characteristics of participants by treatment group. Overall, most participants were women (65 %) and white (92 %) with a mean (SD) age of 46.8 (12.5) years. All participants were classified with neck pain consistent with category 2 (radiation to the proximal extremities) or 3 (radiation to distal extremities) of the Quebec Task Force for Spinal Disorders [75]. These categories are roughly equivalent to severity grades 1 and 2 proposed by the Neck Pain Task Force for patients with neck pain who seek healthcare [76]. Most participants (90 %) had received chiropractic care in the past, although only 25 % of the sample considered themselves as someone who regularly receives chiropractic care.

**Table 2:** Participant characteristics at baseline

Characteristic	Low Force		Medium Force		High Force	
	(n = 16)		(n = 16)		(n = 16)	
Age in years, mean (SD)	42.2	11.2	47.0	11.4	51.2	13.7
Female, n (%)	11	69	12	75	8	50
White, n (%)	15	94	14	88	15	94
Married, n (%)	7	44	10	63	9	56
Education, high school graduate or higher, n (%)	16	100	16	100	15	94
Employment, full-time, n (%)	11	69	9	56	8	50
Previous chiropractic, n (%)	16	100	14	88	13	81
Regular chiropractic care, n (%)	3	19	5	31	4	25
Body mass index, mean (SD)	31.3	6.9	30.3	6.3	26.9	5.3
Beck depression index, mean (SD)	5	3.8	5	5.0	4	3.7
Current neck pain, Visual Analog Scale (0-100 mm), mean (SD)	45.2	18.0	44.3	14.1	34.5	12.3
Neck disability index (0-50), mean (SD)	10.8	4.0	11.1	4.5	11.1	3.9
Neck pain or symptom radiation pattern (may indicate more than 1 location of symptom radiation)						
Head, n (%)	1	6	2	13	2	13
Thoracic, n (%)	3	19	2	13	4	25
Shoulder, n (%)	8	50	3	19	8	50
Upper arm, n (%)	1	6	0	0	2	13
Distal arm/hand, n (%)	3	19	8	50	2	13
Quebec Task Force Classification						
QTF 2, n (%)	11	23	11	23	14	29
QTF 3, n (%)	5	10	5	10	2	4
PROMIS-pain interference, mean (SD)	53.2	6.7	54.9	5.2	51.2	5.6
PROMIS-physical function score, mean (SD)	27.7	5.2	25.3	5.0	26.4	4.5
PROMIS-sleep disturbance, mean (SD)	51.3	3.0	51.3	3.0	51.5	2.6
PROMIS-fatigue, mean (SD)	50.4	10.9	46.4	9.3	47.1	7.2

*PROMIS* Patient-Reported Outcomes Measurement Information System, *QTF 2* Quebec Task Force Rating 2 - pain and radiation to proximal extremity, *QTF 3* Quebec Task Force Rating 3 - pain and radiation to distal extremity

Current neck pain on the VAS at BL1 for the sample was a mean (SD) of 41.3 (15.4) indicating mild neck pain [55]. Participants allocated to the higher force group reported lower levels of current pain at BL1 than participants in the other groups. The NDI scores for the sample measured a mean (SD) of 11.0 (4.1), consistent with a mild disability from neck symptoms [77]. Participants reported a mean duration of neck pain of 14 years, ranging from 1-45 years. Participants’ self-reported cause of neck pain included a gradual onset (n = 17), no known cause (n = 8), office or desk work (n = 7), motor vehicle accidents (n = 6), sports injury (n = 5), manual labor (n = 3) or an accidental fall (n = 2). Participants reported a mean of 2.5 (range 0 to 7) neck pain aggravators, with frequent aggravators noted as seated posture (n = 18), reading (n = 15), computer use (n = 13), sleep position or quality (n = 9), driving (n = 8), neck or head motions (n = 8), lifting (n = 8), stress (n = 8), motor vehicle accidents (n = 6), sports participation or injuries (n = 6), housework (n = 5), office or desk work (n = 4), standing posture (n = 2), weather change (n = 2), music playing (n = 2), and other aggravators (n = 8).

### Patient-reported outcomes

Pain intensity at baseline was significant (p = 0.07) in the model for change in pain interference and, therefore, remained in the final reported model. The traction-force feedback method was significant in the models for change in VAS (p = 0.06) and change in NDI (p = 0.01), and remained in the final reported models. Sex and BMI were not significant in any of the models.

#### Neck pain intensity

**Fig. 4:**
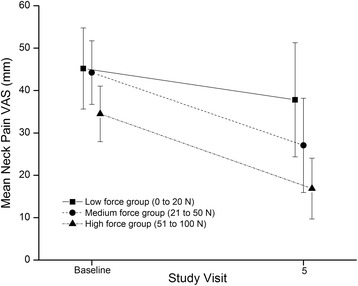
Pain VAS for the three intervention groups at baseline and study visit 5. *VAS* visual analogue scale

The raw mean VAS at baseline and SV5 for each group is given in Fig. 4. Adjusted mean neck pain VAS change (95 % confidence interval) was 9.1 mm (-1.6 to 19.8 mm), 18.9 mm (7.7 to 30.1 mm), and 24.7 mm (13.9 to 35.5 mm) for the low, medium and high force groups, respectively. The adjusted mean neck pain intensity change for the high traction force MCD group was significantly greater (15.6 mm) than the low force group (Table 3). The adjusted mean neck pain intensity change was 9.8 mm greater for those in the medium traction force MCD than those in the low force group, but there was little difference compared to the high force group (5.8 mm).

**Table 3:** Adjusted mean differences of patient-reported outcomes

Outcome variable	Treatment group comparison	Mean difference^a^	95 % Confidence interval^a^
Neck Pain VAS (mm)^b^	High vs. Low	15.6	1.6 to 29.7
	Medium vs. Low	9.8	−3.7 to 23.3
	High vs. Medium	5.8	−8.6 to 20.3
Neck Disability Index^b^	High vs. Low	2.7	−0.1 to 5.6
	Medium vs. Low	3.0	0.1 to 5.9
	High vs. Medium	0.2	−2.7 to 3.2
PROMIS –			
Pain Interference^c^	High vs. Low	3.9	−5.6 to 2.7
	Medium vs. Low	−1.5	1.0 to 0.1
	High vs. Medium	5.4	−2.2 to 9.8
PROMIS –			
Physical Function	High vs. Low	−0.2	−2.2 to 1.9
	Medium vs. Low	−1.0	−3.1 to 1.2
	High vs. Medium	0.3	−1.9 to 2.5
PROMIS - Fatigue	High vs. Low	5.8	1.1 to 10.5
	Medium vs. Low	3.3	−1.5 to 8.2
	High vs. Medium	2.5	−2.3 to 7.3
PROMIS –			
Sleep Disturbance	High vs. Low	1.3	−0.9 to 3.5
	Medium vs. Low	−1.4	−3.6 to 0.9
	High vs. Medium	2.7	0.4 to 5.0

*High* High force group (51 to 100 Newtons), *Low* Low force group (0 to 20 Newtons), *Medium* Medium force group (21 to 50 Newtons), *PROMIS* Patient-Reported Outcomes Measurement Information System, *VAS* Visual Analog Scale

^a^Adjusted for the baseline value of the respective variable centered at its mean;

^b^Also adjusted for traction-force feedback method; ^c^Also adjusted for baseline neck pain VAS

#### Neck-related disability

**Fig. 5:**
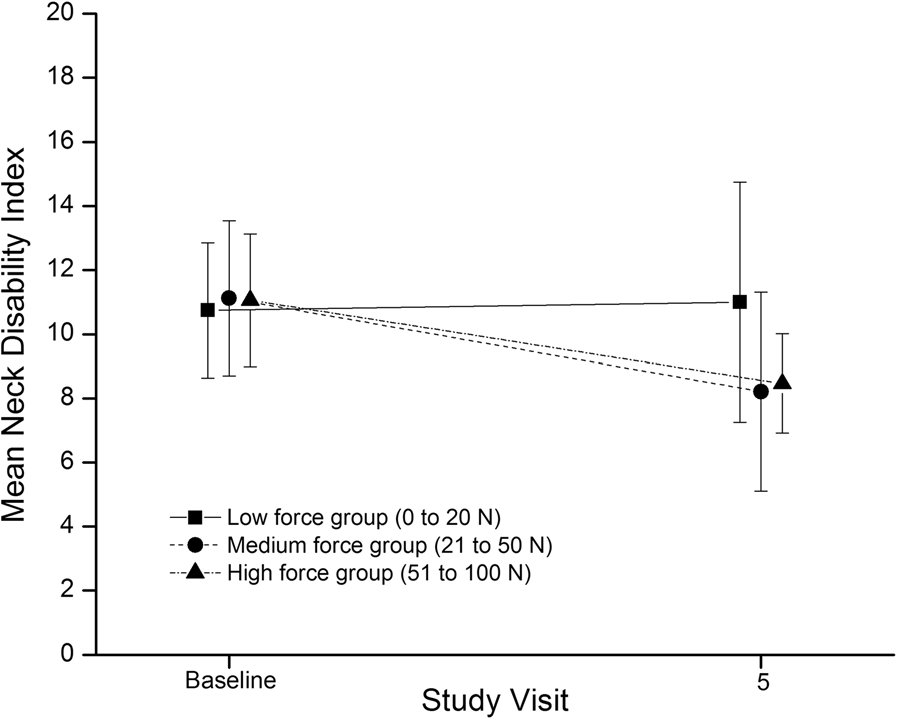
NDI for the three intervention groups at baseline and study visit 5. *NDI* Neck Disability Index

The raw mean NDI at baseline and SV5 for each group is given in Fig. 5. Adjusted mean NDI change (95 % confidence interval) was 1.3 (-1.0 to 3.5), 4.2 (1.8 to 6.6), and 4.0 (1.8 to 6.2) for the low, medium and high force groups, respectively. The adjusted mean NDI change was approximately the same for the medium and high force groups, and both had significantly more change (2.7-3.0 points) than the low force group (Table 3).

#### PROMIS-43 outcomes

Adjusted mean differences on the PROMIS-pain interference and physical function were not appreciably different between groups (Table 3). However, the mean PROMIS fatigue change score was significantly greater for the high traction force MCD than the low force group (5.8).

#### Satisfaction

More high traction force participants (n = 15) were satisfied with the treatments they received compared to participants in the medium (n = 9) or low traction force (n = 7) groups. In contrast, participants in all three groups were satisfied with the clinician who provided their care (high force group, n = 15; medium force group, n = 14; low force group, n = 14).

### Cervical traction forces

Table 4 presents the cervical traction forces delivered to participants. The mean values are within the prescribed force ranges for each group at both the C5 and occiput levels. Maximum forces were highest at the occiput level for all three force groups. Maximum values for the light force group reached 27 N. Minimum values for the medium force group were as low as 8 N and as high as 70 N for the maximum values. Minimum force values for the high force group were 31 N and maximum force was 99 N. In some cases, clinicians delivered MCD cycles outside the allocated force range.

**Table 4:** Cervical traction forces measured in Newtons (N) over four treatment visits

Force range	Contact level	Number of observations^a^	Mean	SD	Minimum	Maximum
Light Force (0-20 N)	C5	64	13.82	5.74	0.18	23.31
	Occiput	64	17.19	4.84	0.47	26.64
Medium Force (21-50 N)	C5	57	38.30	12.12	8.61	59.07
	Occiput	56	42.87	10.40	7.70	69.57
High Force (51-100 N)	C5	60	65.08	17.70	30.63	89.65
	Occiput	60	74.06	16.08	37.54	98.34

^a^We recorded 23 missing values for traction forces due to 9 missed appointments (18 observations) and 5 instances of technical problems in data collection

### Treatment credibility and expectancy measures

Table 5 depicts the changes in CEQ scores between treatment groups over the two-week trial. Credibility factor scores at BL1 suggest participants considered MCD, as described in the consent document and introductory video, to be a credible therapy for neck pain. After receiving MCD for the first time (SV1), participants in the high force group reported higher credibility scores from BL1, while credibility scores fell for medium and low force group participants. Credibility score trends continued at the final visit, where the high force group reported the highest credibility scores, the medium force group remained stable, and the low force group dropped six points from BL1. Expectancy factor scores followed similar patterns. Participants in the high force group reported greater expectancy scores after receiving MCD at SV1 compared to the other groups. However, expectancy scorings dropped for all treatment groups at SV5.

**Table 5:** Credibility and expectancy questionnaire results

	Baseline 1		Study visit 1		Study visit 5	
	Mean	SD	Mean	SD	Mean	SD
Credibility factor						
Low force	19.8	2.2	15.5	7.0	13.8	8.0
Medium force	23.1	3.4	19.9	5.2	19.9	7.5
High force	21.0	3.9	22.1	4.8	22.7	4.0
Expectancy factor						
Low force	15.8	4.0	13.4	6.2	11.4	7.8
Medium force	19.7	4.7	17.8	5.8	15.1	8.3
High force	16.0	6.9	19.3	6.7	17.8	6.2

### Qualitative analysis of treatment group allocation

Thirty-nine (81 %) participants completed a qualitative interview which included two questions that asked participants to identify their treatment group assignment. No interviews were conducted with the three participants who withdrew from the study (two medium force, one high force) while scheduling conflicts prevented the completion of six additional interviews (three low force, two medium force, one high force). We completed interviews with the majority of participants in each treatment group. With this number of interviews, we achieved consistent thematic coding of participants’ attributions for treatment allocation within and across groups (largely related to the amount of pressure or force used by clinician during treatment delivery), suggesting we reached theoretical saturation on this topic [78].

In the low force group, 12 of 13 (92 %) participants correctly identified their treatment assignment as ‘low force’. Most participants noted the light touch used by the clinician as the reason for their treatment group attribution, as indicated by this participant: “It didn’t feel like there was much pressure being put on there.” One low force participant incorrectly identified the treatment allocation stating, “I believe I received the medium, but I’m not sure. It wasn’t really tight, but it wasn’t really soft either. It was sort of like an in-between.” In the medium force group, 4 of 13 (31 %) participants correctly identified their treatment assignment as ‘medium force’, with 7 participants incorrectly guessing their group (6 as low force, 1 as high force), and 2 participants identifying their group as ‘low-to-medium’ force. In the high force group, 2 of 13 (15 %) participants correctly identified their group allocation as high force, while 9 (69 %) participants incorrectly guessed their group (8 as medium force, 1 as low force), and 2 participants assessed that they were in a ‘medium-to-high’ force treatment group. The participants who correctly identified their treatment group as high force noted the clinicians’ behavior as an indicator of their assignment, such as, “He (the chiropractor) seemed to exert himself a little bit”. In contrast, high force participants who incorrectly guessed their group assignment expected more forceful sensations from the treatment, as did this participant: “He (the chiropractor) could have pushed harder, you know?”

### Participant safety

No serious adverse events were documented. Fifty-seven adverse events were reported by 29 participants (including 1 non-randomized). However, 34 of the AEs reported by 23 participants were graded as unrelated (n = 30) or unlikely (n = 4) related to study interventions and were classified as mild (n = 32) or moderate (n = 2) in severity. Another 23 AEs were reported by 14 participants and graded as possibly (n = 1), probably (n = 6) or definitely (n = 15) related to study interventions. These related AEs were all classified as mild severity (that is, transient and minimal symptoms needing no change in activity level or additional treatment) and were characterized as neck or back soreness or pain (n = 14), muscle or joint stiffness (n = 3), combined pain and stiffness (n = 2), headache (n = 1), vertigo (n = 1), and pain paired with a flushed sensation (n = 1). One episode of vertigo that occurred within 30 hours of SV1 in a participant with previous episodes of dizziness was classified as a severe AE that was probably related to the study interventions. Of the 23 AEs attributed to the study, 3 AEs were reported by 3 participants assigned to the low force group, 15 AEs were reported by 6 participants assigned to the medium force group, and 5 AEs were reported by 5 participants assigned to the high force group. Twenty randomized participants reported no adverse events, including nine low force, five medium force, and six high force participants.

### Protocol deviation

We recorded one protocol deviation in this trial. Our original plan was to train research clinicians to deliver MCD at the three traction force ranges using tactile sensations alone, as manual therapists deliver treatments in clinical practice. During a monthly re-certification, investigators noted modest drift in the clinicians’ delivery of MCD. Delivery of the low force group drifted into the medium range, particularly for forces delivered to the occiput, although traction forces remained under 25 N. Medium traction forces drifted in each direction and at both contact levels. High traction forces drifted toward the medium range, for both vertebral levels, with most drift measured between 30 N to 50 N; no traction forces were noted above 100 N. To address this drift, the researchers developed a visual feedback tool to augment the auditory and graphical feedback received by clinicians during training (Motion Monitor software, Version 7.1, Innovative Sports Training, Inc.). The instrument allowed clinicians to view a computer monitor displaying visual force-feedback information in real time while performing MCD. The monitor displayed a cursor which represented traction force. The visual feedback tool was implemented in the RCT on 27 June 2013. Clinicians provided a total of 231 MCD treatments without visual feedback and 218 treatments with visual feedback. We reported the impact of this protocol change on the proficiency of clinician traction force delivery elsewhere [45].

## Discussion

This paper presents the results of a pilot randomized clinical trial of MCD for patients with chronic neck pain designed to evaluate an attention-touch control for future clinical trials. MCD is a traction-based treatment for cervical and thoracic spine pain and dysfunction that is commonly delivered by DCs in the USA [79]. Potential advantages of MCD over other types of traction include its delivery in the prone position, which may enhance the localization of manual forces applied more specifically to a region of the spine compared with other treatments that apply traction via straps around the mandible and occipital region of the head. To our knowledge, no other studies have investigated MCD using a force-based approach or assessed the traction forces delivered during MCD using biomechanical measures. In addition, the technological advancement represented by real-time force feedback enhances the feasibility of consistent treatment delivery force [46].

One advantage of this study was its mixed methods approach to the development of a minimal intervention control for RCTs of traction-based therapies. Manual therapy research has been hampered by the lack of viable control procedures [32, 80–82]. As manual therapies require physical contact between the clinician and patient, it is difficult to introduce a believable sham or minimal intervention that is not immediately obvious to participants or that has no specific effect on the clinical outcomes under investigation [81]. Vernon and colleagues developed a sham cervical procedure that 50 % of participants suspected was real SM, and that had no clinically important changes in cervical range of motion, pain or tenderness [83]. However, this sham procedure is a high-velocity spinal manipulation maneuver, quite unlike the gradual spinal mobilization of MCD, making it ill-suited as a control intervention for studies of traction procedures.

We developed a traction force-based minimal intervention that demonstrated some characteristics of a successful attention-touch control group, such as the lack of clinically important changes in the primary outcomes. Participants who received the low force treatment did identify this procedure as the minimal intervention and stated their assessment was due to the lightness of the clinician’s touch. Further, many medium force participants incorrectly guessed that they had received the low force intervention. As our team measured the amount of traction force participants received at each treatment visit, it was possible to identify the mean traction forces delivered to each group. Based on these combined qualitative and traction force data, we will modify the force ranges for the minimal intervention and MCD groups in future studies. An increase in the minimal intervention traction force range from 0 to 20 N to 15 to 30 N should enhance treatment credibility (that is, the lightness of the clinician’s touch) while still minimizing the treatment effect. In this pilot, the traction force range for the high force group was 51 to 100 N. To maximize the distinction between a minimal intervention and the MCD group in future trials, we will set the MCD traction force range at 60 to 100 N, which is consistent with the peak forces measured in the high force group, as well as participants’ qualitative assessments of treatment delivery in this group. Future trials should conduct similar qualitative assessment to ascertain whether these modifications adequately enhance the credibility of the minimal intervention control.

The changes in patient-reported outcomes during this pilot were promising. Group differences were demonstrated on the neck pain VAS and NDI for the medium force and high force groups compared to the low force group. Unexpected, but intriguing changes were the group differences noted in fatigue and sleep disturbance outcomes. However, differences in the primary and secondary outcomes between the medium and high force groups were negligible. In part, this finding may be due the overlap in traction forces between groups noted midway through the trial which was resolved by the use of visual force feedback technology [46]. Future studies of MCD might opt to compare treatment groups with more distinct differences such as Snodgrass et al. reported in a pilot study utilizing a force-based posterior-to-anterior mobilization procedure [84]. In that study, significant reductions in pain and spinal stiffness were reported in participants receiving the high force posterior-to-anterior mobilization (90 N) compared to low force (30 N) or placebo [84]. As the greatest improvements in neck pain severity and neck-related disability in our study were reported by the high traction force group, a fully powered future RCT might compare high force traction to a minimal intervention, attention-touch control.

Of note, the improvements in PROs reported in this study were achieved in a pilot RCT that was only two weeks in duration. Future RCTs that evaluate MCD might extend the course of care to four or six weeks, as is delivered in clinical practice [34] and in recent clinical trials of traction-based therapies which demonstrated both short-term and longer term improvements in neck pain intensity and disability following four weeks of treatment [24, 85].

Additional aims of this pilot study were to assess the feasibility of the participant recruitment plan and to evaluate the safety of force-based delivery of MCD. Community interest in this trial was good as evidenced by the consistent participant recruitment, enrollment and trial completion. We enrolled a middle-aged sample with the majority of participants being women, which is consistent with the patient population that reports musculoskeletal neck pain [7, 8]. Data collection took nine months, nearly three months sooner than anticipated during study development. Few participants missed any visits, only three participants withdrew from the trial, and none were from the low force group. These results support the feasibility of our participant recruitment plan for a fully-powered randomized controlled trial. Additional recruitment methods that might enhance enrollment during a larger trial are outreach to community groups, press releases and social media announcements, particularly those targeting populations in which neck pain is a prominent concern such as middle aged women, as well as the possible addition of financial incentives [49, 86]. We randomized participants who reported symptoms consistent with QTF classifications 2 (neck pain with radiation to proximal extremities) and 3 (neck pain with radiation to distal extremities). In future trials of MCD, researchers may consider partnering with healthcare providers who treat persons with chronic neck pain to identify potential participants with these clinical characteristics.

During our pilot, we limited our inclusion criteria to participants who rated their neck pain at baseline as between 3 and 7 on the NRS as a safety precaution. Thus, a number of potential participants (n = 32) were excluded for pain severity levels of 8, 9 or 10 on the NRS. Given the relatively low number of adverse events related to study interventions reported in the trial, which were equally distributed between groups, of mild or moderate severity, and followed established patterns of adverse events from spinal mobilization reported in the literature [69, 73, 74, 87], opening eligibility criteria to include persons with greater neck pain severity levels seems acceptable and would likely increase participant eligibility for a larger trial.

This pilot RCT had several limitations. As in manual therapy trials generally [88], the clinicians who delivered the intervention were not blinded to treatment group nor were the biomechanics research assistants blinded to force measurements. Both types of personnel received extensive training and followed an explicit script to prevent the inadvertent unblinding of study participants to their treatment group. In addition, study participants were excluded if they reported high levels of neck pain, cervical fusion, and several other co-morbid conditions which are common among persons with chronic neck pain [2]. No additional measures were employed to control for the angle of distraction, which theoretically varies between participants who inherently exhibit varied morphology. Characteristics affecting the angle of distraction could include thoracic cage diameter, breast tissue, abdominal tissue, and cervico-thoracic curvature.

Our study tested a single component of MCD – traction in a neutral head position – which is thought to be the most important component contributing therapeutic benefit [34]. However, practitioners who use MCD often incorporate multiple manually delivered therapies focused on the cervical and thoracic spine into their treatment regimens. In addition, the manual contact points treated in clinical practice may differ from those in this study (C5, occiput). Also, the number of treatments provided (n = 5) is potentially dissimilar to the use of MCD in practice settings, in that the clinician determines the frequency and duration of treatment based on the type and severity of the condition, the patient’s response to treatment, and the patient’s care phase (acute, subacute, chronic, rehabilitation) [34]. Our decision to standardize these delivery components of the MCD intervention increased the rigor of this pilot RCT, but may have limited the participants’ clinical response to the intervention. If the efficacy of traction with a neutral head position is established, trials to assess the full complement of the MCD intervention may be warranted.

Another limitation is the cost of the equipment used to measure traction forces. Gudavalli et al. reported on the proficiency of trained DCs demonstrating 75 % proficiency with no feedback and 97 % with visual feedback [46]. The technology used in this study was developed for research purposes rather than clinical application. This equipment may be cost prohibitive for many clinicians to purchase for their practice settings. The development of a low cost technology to offer force feedback in practice settings is feasible and would advance the training of manual therapists.

## Conclusions

This paper reports the primary findings of a pilot randomized clinical trial of MCD for adult patients with chronic neck pain designed to develop a minimal intervention to serve as an attention-touch control for future studies. Participants who received the medium (21 N to 50 N) to high (51 N to 100 N) traction force MCD reported clinically important improvements in neck pain intensity and neck-related disability compared to participants who received low (≤20 N) traction force MCD. These improvements in clinical outcomes were noted after only four sessions of MCD delivered over a two-week period. Adverse events were of mild or moderate severity and self-resolved, demonstrating the safety of the intervention. These data support undertaking a fully powered, randomized controlled trial of MCD using the minimal intervention as an attention-touch control.

### Consent to use photographs

Written informed consent was obtained from the patient(s) for publication of this manuscript and accompanying images. A copy of the written consent is available for review by the Editor-in-Chief of this journal.

## Abbreviations

AE: Adverse event
BL1: Baseline 1 visit
BL2: Baseline 2 visit
C5: 5th Cervical vertebra
CEQ: Credibility and expectancy questionnaire
Hz: Hertz
IMMPACT: Initiative on methods, measurement, and pain assessment in clinical trials
MCD: Manual cervical distraction
N: Newton
NDI: Neck disability index
NRS: Numerical rating scale
PROMIS: Patient reported outcomes measurement information system
RCT: Randomized clinical trial
SAE: Serious adverse event
SAS: SAS Institute, Inc.
SQL: Structured query language
SSL: Secure socket layers
SV: Study visit
VAS: Visual analogue scale
